# Significance of EZH2 expression in canine mammary tumors

**DOI:** 10.1186/s12917-016-0789-2

**Published:** 2016-08-09

**Authors:** Hyun-Ji Choi, Sungwoong Jang, Jae-Eun Ryu, Hyo-Ju Lee, Han-Byul Lee, Woo-Sung Ahn, Hye-Jin Kim, Hyo-Jin Lee, Hee Jin Lee, Gyung-Yub Gong, Woo-Chan Son

**Affiliations:** 1 Asan Institute for Life Sciences, Asan Medical Center, Seoul, Republic of Korea; 2 Department of Pathology, University of Ulsan College of Medicine, Asan Medical Center, 88 Olympic-ro 43-gil, Songpa-gu, Seoul, 138-736 South Korea

**Keywords:** Dog, Canine mammary tumor, EZH2, Comparative oncology

## Abstract

**Background:**

Current studies report that aberrations in epigenetic regulators or chromatin modifications are related to tumor development and maintenance. EZH2 (Enhancer of zeste homolog 2) is one of the catalytic subunits of Polycomb repressive complex 2, a crucial epigenetic regulator. EZH2 has a master regulatory function in such processes as cell proliferation, stem cell differentiation, and early embryogenesis. In humans, EZH2 is linked to oncogenic function in several carcinomas, including breast cancer, and dysregulation of EZH2 has been particularly associated with loss of differentiation and the development of poorly differentiated breast cancer. In our present study, we were interested in determining whether EZH2 is increased in canine mammary tumors, which show similarities to human breast cancer.

**Results:**

Investigation of the expression of EZH2 in canine mammary tumors revealed that EZH2 protein was overexpressed in canine mammary carcinomas, as in human breast cancer. In addition, the immunohistochemical expression level of EZH2 was associated with the degree of malignancy in canine mammary carcinoma. This is the first report to describe EZH2 expression in canine mammary tumors.

**Conclusions:**

Because the expression of EZH2 was similar in canine mammary carcinoma and human breast cancer, spontaneous canine mammary tumors may be a suitable model for studying EZH2 and treatment development.

## Background

Breast cancer is expected to be the most common malignancy among women in the United States in 2015 [1]. Although several prognostic markers in human breast cancer have been investigated, only a small number of markers are in clinical use, possibly because of a poor correlation between the findings of animal studies and clinical trials. Therefore, there is a need for more appropriate therapeutic targets and additional models to improve the understanding and biological characterization of human breast cancer and therapeutic development.

EZH2 is a catalytic subunit of the epigenetic regulator Polycomb repressive complex 2 (PRC2). PRC2, which includes EZH2, suppressor of zeste 12 (SUZ12), and embryonic ectoderm development (EED), trimethylates histone 3 lysine residue 27 (H3K27) and leads to silencing of genes involved in processes such as stem cell maintenance and tumor progression without DNA sequence modification [2–4]. Recently, overexpression or mutation of EZH2 have been found in a wide range of human tumors, including breast, prostate, urinary bladder, ovarian, lung, gastric cancer, renal cell carcinoma, and glioblastoma [5–12]. The evidence indicates that EZH2 plays a role in the initiation, development, progression, and metastasis of cancer and drug resistance [13]. In particular, EZH2 has been connected to the aggressiveness of breast cancer [14, 15]. In addition, EZH2 has been reported to be an adverse prognostic marker for breast cancer and an index of an unfavorable tamoxifen outcome [16–18]. A correlation between loss of differentiation and deregulated expression of EZH2 has also been proposed in human breast cancer [19]. Recent evidence implicates EZH2 in transcriptional activation, but the mechanisms are not clearly defined [20].

In order to investigate the feasibility of an additional animal model to improve our understanding of EZH2, we collected and investigated naturally occurring canine mammary tumors (CMTs). Mammary tumors are the most commonly diagnosed neoplasms in female dogs and nearly 50 % are malignant [21, 22]. One study has found an annual incidence of mammary tumors of 16.8–47.7 % (benign) and 47.5 % (malignant) [23]. This incidence is greater than that of human breast cancers. Dog and human lineages are similar in terms of both nucleotide divergence and rearrangements [24] and dogs have been suggested as additional tumor models [25, 26]. Although histomorphological features may differ between human breast cancer and CMTs, they share many similarities in terms of age of onset, risk factors, molecular marker expression, behavior, and prognosis [27–30]. In addition, the incidence of CMTs is sufficiently high to secure proper number of subjects in clinical trials and the size of dogs makes multimodality protocols feasible [31]. Therefore, it is expected that the study of cancer using mammary tumors of domesticated dog might provide new insights into cancer understanding and therapy development.

The purpose of our present study was to investigate EZH2 in CMTs. We found that EZH2 is overexpressed in clinical samples of canine mammary carcinomas.

## Results

### Histological evaluation

**Fig. 1:**
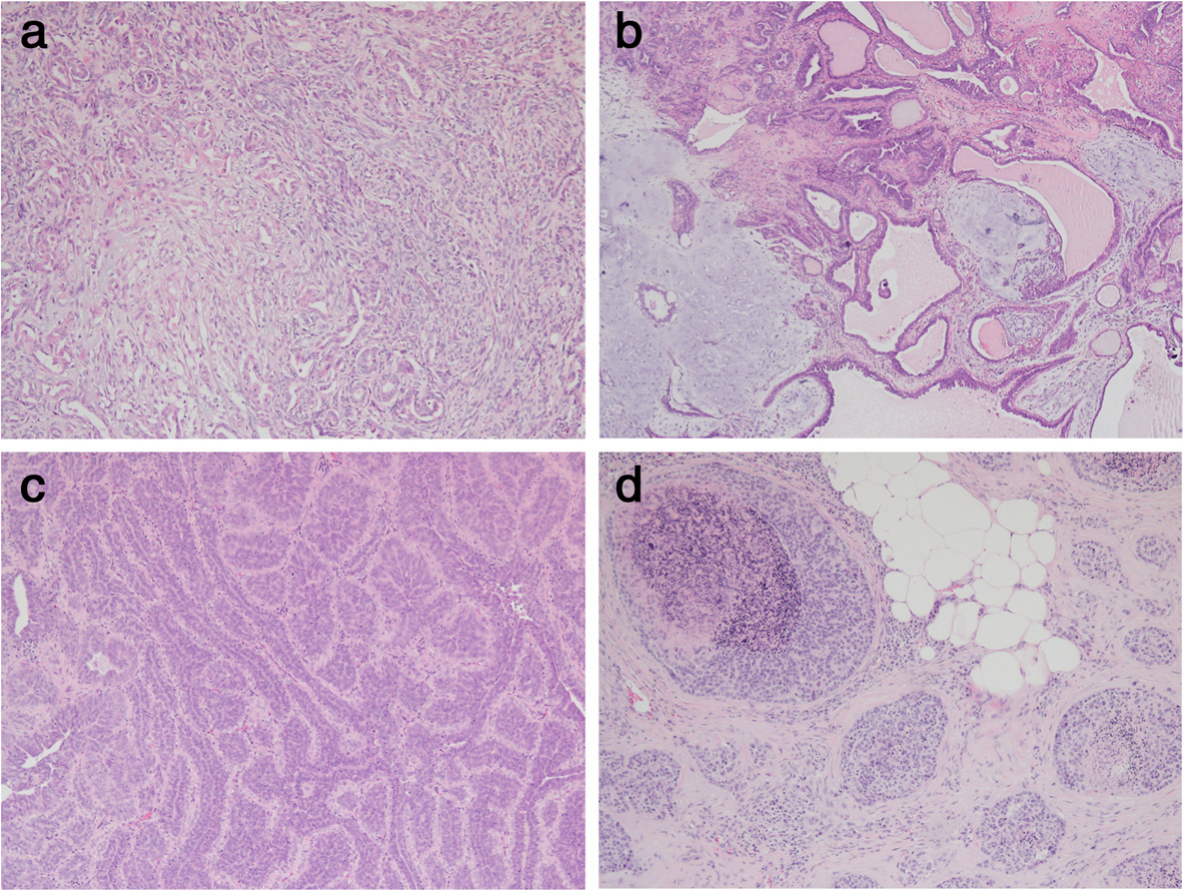
Representative mammary carcinoma tissues with H&E staining. **a** Carcinoma complex type with a malignant epithelial component and a benign myoepithelial component. **b** Carcinoma mixed type with a malignant epithelial component and a benign mesenchymal component (cartilage). **c** Carcinoma, tubular type. The tumor cells are predominantly arranged in a tubular pattern. **d** Comedocarcinoma. There are necrotic areas within the center of neoplastic cell aggregates

The clinical and morphologic features of the 74 mammary gland cases were identified (Table 1). There were five non-neoplastic lesions including lobular hyperplasia and duct ectasia. Sixty-nine CMT cases showed benign morphological features (3 cases, 4 %) and malignant features (66 cases, 96 %) such as carcinoma with simple tubular or tubulopapillary type, complex type, mixed type, solid type, anaplastic and inflammatory carcinoma, mucinous carcinoma, lipid-rich carcinoma, and comedocarcinoma (Fig. 1). The most common type of carcinoma identified was the complex type (26 cases, 39 %), followed by mixed type (18 cases, 27 %) and simple tubular type (12 cases, 18 %). Canine mammary carcinomas exhibited a malignancy ranging from 1–3 as follows: 1 (38 cases, 58 %), 2 (18 cases, 27 %), and 3 (10 cases, 15 %).

**Table 1:** Demographics of canine mammary cases

Type	Non-neoplastic lesion			Benign tumor	Malignant tumor					
	Hyperplasia	Ectasia	Total	Adenoma	Complex type	Mixed type	Tubular type	Anaplastic carcinoma	Comedo-carcinoma	Total
Total	4	1	5	3	26	18	12	3	2	61
Mean age, year	8	14	9	6	11	10	10	12	10	11
Malignancy grade, no.										
1	-	-	-	-	15	15	7	0	0	37
2	-	-	-	-	9	1	4	1	0	15
3	-	-	-	-	2	2	1	2	2	9
Mean	-	-	-	-	1.5	1.3	1.5	2.7	3	1.5
EZH2 score, no.										
0	1	1	2	0	0	1	0	0	0	1
1	2	0	2	2	8	9	4	0	0	21
2	1	0	1	1	12	6	4	2	0	24
3	0	0	0	0	6	2	4	1	2	15
Mean	1	0	0.8	1.3	1.9	1.5	2	2.3	3	1.9

### Immunohistochemistry

**Fig. 2:**
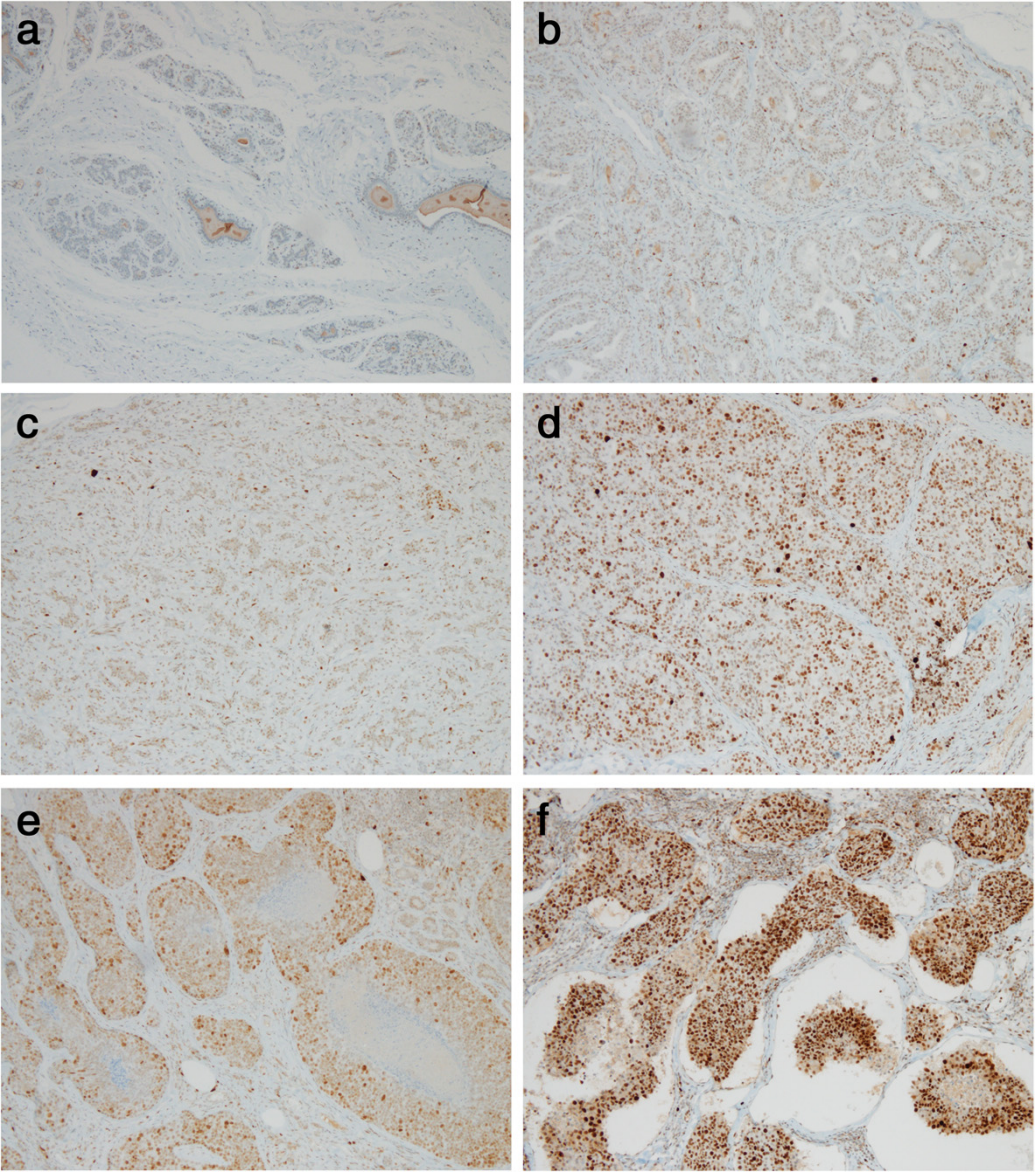
Immunohistchemistry for EZH2. **a** Normal mammary gland. Immunohistchemistry for EZH2 showing negative staining of the mammary gland. **b** Adenoma. The intensity score of EZH2 is 1. **c** Carcinoma mixed type with malignancy grade 1. The intensity score of EZH2 is 1. **d** Carcinoma solid type with malignancy grade 3. Diffuse nuclear staining with the intensity score 3 of EZH2. **e** Comedocarcinoma. The intensity score of EZH2 is 3. **f** Anaplastic carcinoma. Note the strong nuclear staining of neoplastic cells with intensity score 3 of EZH2

**Fig. 3:**
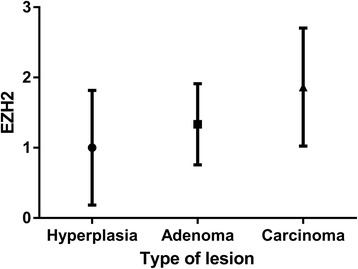
Bar graph of the immunohistochemical intensity scores of EZH2. Carcinoma demonstrated higher expression level of EZH2 than hyperplasia lesion (*P < 0.05)

**Fig. 4:**
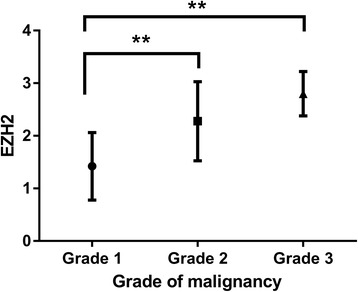
Bar graph of the immunohistochemical intensity scores of EZH2. High malignancy grade tumor showed higher expression level of EZH2 than low grade tumor (**P < 0.01)

**Fig. 5:**
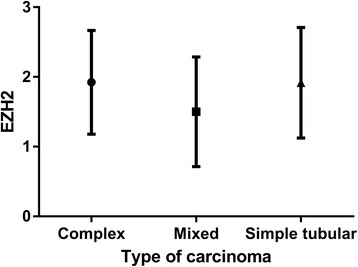
Bar graph of the immunohistochemical intensity scores of EZH2. There is no significant difference between three types of carcinoma

We were interested in determining whether EZH2 is dysregulated in CMTs, which are similar to human breast cancers. In immunohistochemical analyses, there was negative or weak nuclear staining in normal (Fig. 2a) and non-neoplastic mammary tissues. Clear nuclear staining for EZH2 could be observed in CMTs (Fig. 2b-f). The intensity of nuclear pattern was especially strong in comedocarcinoma (Fig. 2e), anaplastic carcinoma (Fig. 2f), and solid carcinoma. Carcinomas showed higher EZH2 expression level than hyperplastic lesions (Fig. 3). High expression of EZH2 was found to be associated with mammary carcinoma malignancy (Table 2, Fig. 4). We sorted each grade of carcinomas according to the expression level of EZH2 to clarify the association between grade of malignancy and EZH2 expression. Malignancy grade 1 was found in 2 cases (5 %) of EZH2 score 0,19 (50 %) of EZH2 score 1, 16 (42 %) of EZH2 score 2, and 1 (3 %) of EZH2 score 3. Malignancy grade 2 was seen in 3 cases (17 %) of EZH2 score 1, 7 (39 %) of EZH2 score 2, and 8 (44 %) of EZH2 score 3. Malignancy grade 3 was present in no cases of EZH2 score 1, 2 (20 %) of EZH2 score 2, and 8 (80 %) of EZH2 score 3. According to these criteria, 99 % cases of CMTs had elevated EZH2 expression. It did not show any relationship within the most frequent three carcinoma types (Complex, mixed and tubular type) of this study (Fig. 5).

**Table 2:** Correlation between malignancy grade and EZH2 score

Malignancy grade	EZH2 score
1	1.4 ± 0.64
2	2.3 ± 0.77
3	2.8 ± 0.42

### Western blotting

**Fig. 6:**
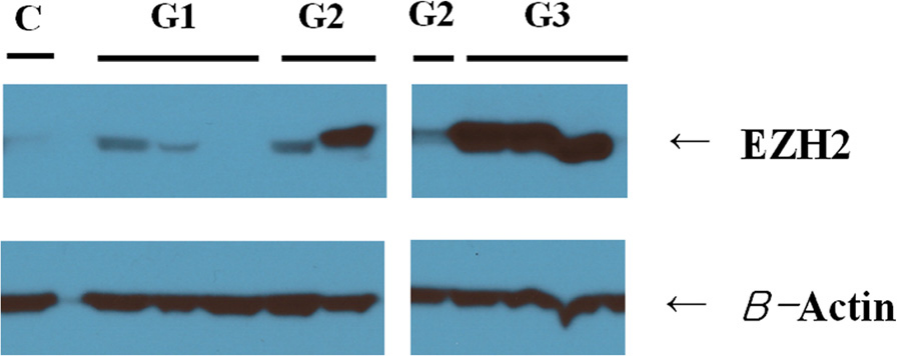
Representative immunoblots of EZH2

We used immunoblotting to compare our immunohistochemistry and molecular data and further investigate the correlations between the grade of malignancy and the expression levels of EZH2. Western blot analysis showed an increase in the expression of EZH2 in the CMT tissues compared with control non-neoplastic mammary tissues. Grade 3 carcinomas showed higher expression of EZH2 than grades 1 and 2 (Fig. 6, Table 3).

**Table 3:** Semi-quantification of western blotting

Group	EZH2/β-actin
Control	1.0
Grade 1	1.4
Grade 2	2.0
Grade 3	2.7

## Discussion

Although increased expression of EZH2 has been observed in aggressive solid tumors in humans, the mechanism involved in the mediation of tumor aggressiveness by EZH2 remains unclear. In our current study, we characterized the expression pattern of EZH2 in CMTs by immunohistochemical staining and immunoblot analysis of CMTs and non-neoplastic mammary tissues. We found that EZH2 protein is increased in CMTs when compared with non-neoplastic mammary tissues. In our immunohistochemical analysis, tumor cells exhibited a clear nuclear staining pattern with various expression levels. When we compared EZH2 expression level with the tumor malignancy grade, type of carcinoma, and type of lesion (hyperplasia/adenoma/carcinoma) respectively, only carcinoma malignancy was found to have statistically significant association with EZH2 expression. The proportion of positive EZH2 staining increased as the malignancy grade increased. Furthermore, there was strong nuclear staining in anaplastic carcinoma, comedocarcinoma which are regarded as high malignant neoplasms between CMTs, and solid carcinoma which reveals poor differentiation. These results are consistent with a previous report that showed a correlation between the EZH2 expression level and aggressiveness or poor differentiation of human breast cancer [15, 19].

We compared the immunohistochemistry results with the immunoblotting results. EZH2 protein showed a higher expression level in grade 3 than grade 1 mammary cancers. There were some results that were inconsistent with the tendency for EZH2 expression to increase with malignancy. First, the number of cases was small and not enough to produce a powerful statistic. We also suspected that one of the reasons for these discrepancies is that there is usually prominent proliferation of the surrounding connective tissue or stroma in CMT tissue. Therefore, homogenization of a small portion of tissue does not always adequately represent the epithelial tumorous component. Nonetheless, there was a tendency for an increased expression of EZH2 with CMT malignancy evident in the immunoblotting results, which was consistent with the immunohistochemistry results. We found no correlation between the carcinoma type and the level of EZH2 expression, possibly due to the relatively small number of samples of each type of carcinoma.

Murine models such as xenograft and transgenic mouse models have been extremely useful tools in the study of human cancer and have provided valuable insights into cancer biology and biochemistry that could not be readily obtained with other models [32]. Despite the importance of these murine models, they have shown a few limitations with respect to some essential features of human cancer, including growth periods, immune function, genomic characterization, and the significant heterogeneity of tumor cells and microenvironments. Even in recently developed patient-derived xenograft models, a higher mutation rate than would arise in the parent tumor, a variable transplantation failure rate, as well as increased costs are major challenges of this approach [33]. Accordingly, dogs with naturally occurring tumors are expected to provide an additional value to researchers.

As the CMTs in our current study in a canine model showed similarities to human breast cancers in terms of EZH2 expression, we suggest that dogs with naturally occurring CMTs could be used as animal models in future clinical trials. Canine tumor models may help to identify novel cancer-associated genes, further elucidate molecular pathways in tumors, and be used in the development of novel diagnostic, prognostic, and therapeutic tools [24].

No EZH2 inhibitors have been approved for the treatment of human cancers to date. The methyltransferase activity of EZH2 is not required for it to activate certain genes. Therefore, approaches based on disrupting the interaction between EZH2 and other factors might be potential therapeutic targets [2]. Because EZH2 plays a diverse role in cancers, insight into the regulation of its signaling would likely aid the development of EZH2-targeted therapeutics. EZH2 inhibitors are currently being developed and clinical trials for B cell lymphoma are ongoing [34, 35].

To our knowledge, our current study is the first to report on the expression of EZH2 in CMTs. Our results lend credence to the view that CMTs are a valuable model for EZH2 studies. However, we have only just begun to understand the biology and functional role of EZH2 in CMTs, and we look forward to studies aimed at further elucidating the mechanisms involved.

## Conclusions

EZH2 is expressed in CMTs, and its levels correlate with carcinoma malignancy.

This leads to a possibility of CMT usage as a model for future EZH2 research and clinical trials on breast cancer.

## Methods

### Tissue samples

Mammary gland samples were collected from 74 female domestic dogs who had been seen at a veterinary clinic due to a mass in the mammary gland. The canine mammary tissues were surgically removed from these animals at a mean age of 12 years (7–15 years) and were submitted to the University of Ulsan College of Medicine between June 2014 and May 2015. After microscopic examination, 5 samples were diagnosed as non-neoplastic lesions and 69 specimens were diagnosed as CMTs.

### Histological evaluation

Slides were evaluated for growth pattern (ductular, papillary, or solid), invasion pattern (expansile, local, regional, nodal, or vascular), mitotic index, degree of necrosis, anaplasia, and inflammation. The morphologic diagnosis of CMT was based on the classification of Goldschidt et al. [36] Using this approach, the classification of benign mammary tumors includes adenoma simple, intraductal papillary adenoma, ductal adenoma, adenoma complex, benign mixed, fibroadenoma, and myoepithelioma. Malignant classifications include carcinoma simple, carcinoma complex, carcinoma mixed, anaplastic carcinoma, lipid-rich carcinoma, inflammatory carcinoma, mucinous carcinoma, and adenosquamous carcinoma. The carcinoma simple class has tubular, tubulopapillary, cystic-papillary, and cribriform subclasses. Tumors that were too poorly differentiated to be morphologically diagnosed were classified as solid carcinomas. Malignancy was evaluated using the following criteria: tumor type, tumor size, tubular formation pattern, significant nuclear and cellular pleomorphism, mitotic index (number of cells with mitotic figures per 10 high-power fields from the neoplastic area with mitotic activity), and presence of areas of necrosis [22]. All samples were also classified according to their morphologic origin. All microscopic evaluations were performed by two veterinary pathologists.

### Immunohistochemistry

Sections (3 μm) from paraffin-embedded tissue blocks of canine mammary gland tumor tissues were mounted on glass slides. Immunohistochemistry was performed using an automated slide preparation system (Benchmark XT; Ventana Medical Systems Inc., Tucson, AZ). Deparaffinization, epitope retrieval, and immunostaining were performed according to the manufacturer’s instructions with cell conditioning solutions (standard, for 60 min) and the BMK ultraVIEW diaminobenzidine detection system (Ventana Medical Systems). Tumor sections were stained with EZH2 (1:100, ab109398, Abcam, Cambridge, MA) for 36 min at 37 °C, followed by Ultraview HRP universal Multimer for 8 min at 37 °C. Positive signals were amplified using ultraVIEW copper, and sections were counterstained with hematoxylin and bluing reagent for 4 min respectively.

### Immunohistochemical Evaluation of EZH2

EZH2 expression was evaluated on the slides using a semiquantitative scoring system described previously with some modifications [10]. Samples were evaluated for staining intensity (0, none; 1, weakly positive; 2, moderately positive; and 3, strongly positive).

### SDS-PAGE and western blot

Approximately 10 mg of CMT tissue were prepared by TissueLyser II (Qiagen, Valencia, CA) and suspended in sample buffer (62 mmol/liter Tris-Cl, pH 6.8, 2 % SDS, 10 % glycerol, and 0.01 % bromophenol blue with 5 % 2-mercaptoethanol), incubated for 5 min at 100 °C, and then electrophoretically separated in a 12 % polyacrylamide mini-gel. Electrophoresis was performed in Tris-buffered saline (TBS) at a constant current of 60 mA for 2 h. Molecular weight standards (P8502-050; GenDEPOT) were run simultaneously. The gel was stained with Coomassie Blue. A parallel SDS-PAGE gel was run as described above, and the separated proteins were transferred directly by tank blotting onto a polyvinyl difluoride transfer membrane (Bio-Rad Corp, Hercules, CA) for 90 min at a constant current of 80 mA. After saturation of the nonspecific sites with 5 % nonfat milk/TBS overnight at 4 °C, the proteins were probed with a 1:500 dilution of rabbit anti-EZH2 antibody (ab186006; Abcam, Cambridge, MA) overnight at 4 °C. The blot was then washed in 20 mM Tris-HCl, pH 7.5, and 0.14 mM NaCl containing 0.5 % Tween 20 (TBS-Tween) and then incubated for 2 h in an anti-rabbit HRP-conjugated IgG antibody (SC-2004; Santa Cruz, Santa Cruz, CA) diluted 1:1000 in TBS-Tween at room temperature. The immunoblot was exposed to an enhanced chemiluminescence immunoassay substrate reagent (DG-WP250; DoGen, Seoul, Korea) for 1 min to detect signals and the membrane was exposed to X-ray film for 5 min. Band intensity on exposed film was semi-quantified using ImageJ software (National Institutes of Health, Bethesda, MD).

### Statistical analysis

Data are expressed as the mean ± standard deviation of the mean. Since our data were not normally distributed in the Kolmogorov-Smirnov test, we compared the data with the Kruskal-Wallis test, which is a non-parametric method using the SPSS version 21 (IBM Corp., Armonk, NY). If significant, paired comparisons were done with the Mann Whitney test. A Bonferronic correction was applied to correct for multiple comparisons of the primary end point.

## Abbreviations

CMTs, canine mammary tumors; EED, embryonic ectoderm development; EZH2, enhancer of zeste homolog 2; H3K27, histone 3 lysine residue 27; PRC2, polycomb repressive complex 2; SUZ12, suppressor of zeste 12
